# Fine-Tuning Positive-Surface-Charge Carbon Dots for High-Efficiency and Low-Cytotoxicity Gene Delivery

**DOI:** 10.3390/nano16030169

**Published:** 2026-01-26

**Authors:** Shuo Zhang, Yangming Zhou, Qi Zhang, Juanjuan Xue, Ruijie Li, Tao Liu, Qianqian Duan, Shengbo Sang

**Affiliations:** Shanxi Key Laboratory of Artificial Intelligence & Micro Nano Sensors, School of Integrated Circuit, Taiyuan University of Technology, Taiyuan 030024, China; zshuo0123@163.com (S.Z.); 2023521518@tyut.edu.cn (Y.Z.); 13593432945@163.com (Q.Z.); xuejuanjuan@tyut.edu.cn (J.X.); 2023521510@tyut.edu.cn (R.L.); wang1577616430@163.com (T.L.)

**Keywords:** carbon dots, gene delivery, surface potential, Ihh

## Abstract

Carbon dots (CDs) have emerged as a promising non-viral gene delivery vector due to their excellent biocompatibility and tunable surface properties. In this study, four CDs with gradient-positive zeta potentials (7.23 mV, 16.7 mV, 25.3 mV, 34.5 mV) were synthesized via a hydrothermal method. Among these, CDs-3 with an optimal zeta potential of 25.3 mV stood out, exhibiting ultra-low cytotoxicity (cell viability > 80% even at 50 μg/mL) and a transfection efficiency of nearly 100% (for GFP plasmid delivery), significantly outperforming commercial vectors Lipo2000 and PEI. A stable CDs-3/siIhh delivery system was constructed at a mass ratio of 2:1. In vitro evaluations confirmed that CDs-3/siIhh could efficiently regulate the Indian Hedgehog (Ihh) signaling pathway and osteoarthritis (OA)-related markers in both normal and IL-1β-induced inflammatory ATDC5 chondrocytes. Its regulatory effect was significantly superior to that of the commercial Lipo2000/siIhh and PEI/siIhh systems. This consistent “transcription–translation” regulation, combined with the carrier’s safety and excellent cellular internalization capacity in chondrocytes, highlights its potential for OA gene therapy. Collectively, our work develops a novel, safe, and efficient positive-potential CD-based gene delivery vector, providing a promising gene regulatory capacity by leveraging optimized surface charge engineering.

## 1. Introduction

Gene therapy represents a transformative approach in modern medicine, holding immense potential for treating a wide range of genetic and acquired diseases by introducing, suppressing, or editing specific genes [1]. Among its various strategies, RNA interference (RNAi) technology, particularly utilizing small interfering RNA (siRNA), has emerged as a powerful tool for sequence-specific silencing of disease-associated genes [2]. The prospect of precisely targeting and downregulating pathogenic genes offers a paradigm shift from conventional symptomatic management to addressing the root molecular causes of diseases. However, the clinical translation of siRNA therapeutics is critically dependent on the development of safe and efficient delivery vectors that can protect the nucleic acid payload, facilitating its cellular internalization [3].

Delivery vectors are broadly categorized into viral and non-viral systems. Viral vectors, such as adenoviruses and lentiviruses, are highly efficient at gene transfer due to their evolved mechanisms for cell entry, but are hampered by significant safety concerns, including immunogenicity, insertional mutagenesis, and limited cargo capacity [4,5]. Consequently, non-viral vectors have garnered substantial interest as safer alternatives. These include cationic lipids (e.g., Lipofectamine 2000) and polymers (e.g., polyethylenimine, PEI), which complex with siRNA through electrostatic interactions [6,7]. Although some non-viral vectors have achieved clinical success, they often suffer from drawbacks such as relatively low transfection efficiency and dose-dependent cytotoxicity [8]. In addition, due to the dense surface structure of cartilage, traditional delivery systems have difficulty penetrating the cartilage surface, resulting in low drug treatment efficiency [9,10]. Therefore, the quest for novel non-viral vectors with an optimal balance of high efficiency, low toxicity, and good tissue penetration remains a paramount research focus.

Carbon dots (CDs), a class of quasi-spherical carbon-based nanomaterials typically less than 10 nm in size, have recently emerged as promising candidates for biomedical applications [11], including bioimaging, drug delivery [12,13,14], and theranostics [15]. Their appeal lies in their excellent biocompatibility, low toxicity, facile synthesis, and ease of surface functionalization [16]. As gene delivery vectors, CDs can be engineered to possess cationic surfaces, enabling them to form stable nanocomplexes with anionic nucleic acids like siRNA by electrostatic interactions; these complexes have been applied in diverse fields such as tumor therapy [17,18], degenerative disease treatment [19], and tissue regeneration [20,21]. The surface charge, commonly quantified by zeta potential, is a critical parameter governing the stability, cellular uptake, and ultimately, the transfection efficiency of these complexes [22,23,24]. For effective gene delivery, a positive zeta potential is generally desirable as it promotes interaction with the negatively charged cell membrane, facilitating endocytosis [25]. Most reported positive potential CDs used for gene delivery exhibit zeta potentials in the low-to-moderate positive range, typically between +10 mV and +30 mV [26]. CDs with zeta potentials in this range often demonstrate a favorable balance, ensuring strong nucleic acid binding while mitigating the cytotoxicity associated with excessively high positive charges [27].

To ensure the positive potential of the CDs’ vector, strategies usually involve modifying anionic or neutral CDs with highly cationic polymers like PEI [28]. Compared to these methods, the direct synthesis of intrinsically positive-potential CDs without modification offers distinct advantages, as it simplifies the preparation process, enhancing batch-to-batch reproducibility and system stability [29]. However, the development of positive-potential CDs for gene therapy is not without challenges. A key issue is optimizing the positive potential, because insufficient charge leads to poor siRNA complexation and low cellular uptake, and excessively high charge may result in increased cytotoxicity and aggregation in biological fluids. Regrettably, existing studies on CDs used as gene vectors have largely been limited to a single zeta potential value, lacking a systematic investigation into how variations in surface charge influence gene delivery performance [30]. Therefore, it is crucial to synthesize CDs with a gradient of positive zeta potentials and, through systematic comparison of their cytotoxicity, nucleic acid complexation capability, and transfection efficiency, elucidate the relationship between surface potential and gene delivery performance, thereby identifying the optimal potential range that achieves a balance between high-efficiency transfection and good biocompatibility [31].

Osteoarthritis (OA) is a highly prevalent and debilitating degenerative joint disorder [32]. Current clinical management strategies are predominantly aimed at symptomatic relief, underscoring the need for novel approaches that can address the underlying molecular pathology of OA. In this context, the Indian Hedgehog (Ihh) signaling pathway has been identified as a key regulator in OA pathogenesis [33]. Silencing Ihh expression in chondrocytes presents a promising therapeutic strategy to halt or reverse the OA disease process by inhibiting this deleterious signaling cascade [34,35].

**Scheme 1:**
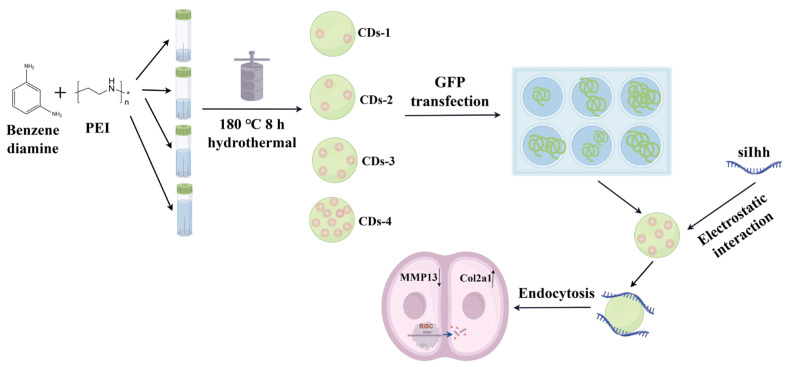
Schematic diagram of the synthesis and application of four carbon dots with different positive zeta potentials. Created with Figdraw.

With the core objective of exploring the optimal positive zeta potential of CDs suitable for efficient gene delivery, this study successfully developed a positive-potential carbon dot-based delivery system targeting the Ihh small interfering RNA (siRNA) (CDs/siIhh). To achieve this goal, four types of CDs with gradient-positive zeta potentials were synthesized via a hydrothermal method by modulating the precursor ratio. Following comprehensive characterization and preliminary evaluations on key parameters for gene delivery such as biocompatibility and transfection efficiency, CDs with the best overall performance were identified as the optimal candidate vector. Based on these optimal CDs, stable CDs/siIhh complex delivery systems were constructed, and in-depth studies were conducted on the biological efficacy of this delivery system, including its cellular internalization capacity and the effect of downregulating the expression of Ihh and its downstream targets at both the transcriptional and translational levels (Scheme 1).

## 2. Materials and Methods

### 2.1. Materials

M-phenylenediamine, polyethyleneimine (PEI 25 KDa), fetal bovine serum (FBS), and high-glucose medium were purchased from Maclin Biochemical Technology (Shanghai, China). Murine chondroblasts (ATDC5 cells) were obtained from the Cell Bank of the Chinese Academy of Sciences (Shanghai, China). Cell Counting Kit-8 (CCK-8) and phosphate-buffered saline (PBS) were bought from Beyotime Biotechnology Co., Ltd. (Shanghai, China). A 0.25% Trypsin-EDTA solution was acquired from Mian Biological Technology (Shanghai, China). The GFP plasmid was purchased from Beyotime Biotechnology with a specification of 2 μg. Small interfering RNA targeting Ihh (siIhh) was synthesized by Boster Biological Technology Co., Ltd. (Wuhan, China) and labeled with the red fluorescent dye Cy5. The mouse siIhh sequence is as follows: sense: 5′-CAG-ACC-GUG-ACC-GAA-AUA-A-3′, antisense: 5′-UUA-UUU-CGG-UCA-CGG-UCU-G-3′ [35]. Interleukin-1β (IL-1β) protein was purchased from Sino Biological Inc (Beijing, China). Primers for quantitative real-time polymerase chain reaction (qPCR) were obtained from Sangon Biotech (Shanghai) Co., Ltd. (Shanghai, China). TRIzol reagent, PrimeScript™ RT Premix, and TB Green™ Premix Ex Taq™ II were all purchased from Takara Biotechnology Co., Ltd. (Otsu, Japan). Lipofectamine 2000 (Lipo2000) was bought from Invitrogen (Carlsbad, CA, USA). RIPA lysis buffer supplemented with protease and phosphatase inhibitors was purchased from Solarbio Co., Ltd. (Beijing, China). Polyvinylidene fluoride (PVDF) membranes were purchased from Nantong Feiyu Biotechnology Co., Ltd. (Nantong, China). TBST buffer was purchased from Jingen Biotechnology (Beijing) Co., Ltd. (Beijing, China). Antibodies against Ihh, Smo, MMP13, Col10a1, Col2a1, and glyceraldehyde-3-phosphate dehydrogenase (GAPDH), as well as the goat anti-rabbit IgG secondary antibody, were purchased from Hangzhou Huidan Biotechnology Co., Ltd. (Hangzhou, China). Deionized water used in the experiment was prepared in the laboratory using an ultrapure water system, UPT-II, manufactured by Shanghai Yiheng Scientific Instrument Co., Ltd. (Shanghai, China). All cell culture consumables were sterilized by high-temperature treatment.

### 2.2. Instruments and Characterization

The morphological characteristics of carbon dots (CDs) were characterized using a high-resolution transmission electron microscope (JEOL Ltd., JEOL 2100 F model, Tokyo, Japan). The surface functional groups of the samples were analyzed by a Fourier transform infrared (FTIR) spectrometer (Bruker Optik, FTIR Tensor 27 model, Ettlingen, Germany). The absorption spectra were recorded with a UV–visible spectrophotometer (Metash, Metash UV-8000A model, Shanghai, China). Meanwhile, the fluorescence spectra were determined using a fluorescence spectrophotometer (Edinburgh Instruments, FLS-980 model, Livingston, UK). X-ray photoelectron spectroscopy (XPS) measurements were performed using an ESCALAB 250xi (Thermo Fisher Scientific Inc., ESCALAB 250xi model, Waltham, MA, USA). The surface charge (zeta potential) of the nanoparticles was analyzed by a nanoparticle size and zeta potential analyzer (Malvern Panalytical, Malvern Zetasizer Nano ZS model, Malvern, UK). The results of agarose gel electrophoresis were visualized on a gel imager (Bio-Rad, MSD-910 A model, Hercules, CA, USA). Cell viability was evaluated using a microplate reader (Thermo Fisher Scientific Inc., Multiskan FC model, Waltham, MA, USA). Representative images of cells were acquired using a Cytation 5 Cell Imaging Multi-Mode Reader (BioTek Instruments, Inc., Winooski, VT, USA). Reverse transcription and gene expression detection were performed using a PCR instrument (Applied Biosystems, Quantstudio^TM^ 6 model, Foster City, CA, USA). The results of western blotting were visualized and analyzed using a gel imaging system (LI-COR Biosciences, Odyssey^®^ CLx model, Lincoln, NE, USA).

### 2.3. Preparation of CDs and CDs/siIhh System

Building on the methodology reported by Zhang et al. [36], four types of carbon dots with tunable positive potentials were synthesized using m-phenylenediamine as the carbon source and PEI as the nitrogen source. The tuning of positive potentials was achieved by adjusting the dosage of PEI. In all synthesis systems, the mass of the carbon source, m-phenylenediamine, was fixed at 300 mg, while the amount of PEI was varied according to predefined molar ratios. Specifically, the molar ratios of the carbon source to the nitrogen source were set as 2:0.5 (CDs-1), 2:0.7 (CDs-2), 2:1 (CDs-3), and 2:1.5 (CDs-4). M-phenylenediamine was dissolved in 20 mL of deionized water, and PEI was dissolved in 10 mL of deionized water. The two solutions mentioned above were mixed and placed in a 50 mL reaction kettle. The reaction kettle was heated to 180 °C in a muffle furnace and maintained at this temperature for 8 h. After the reaction was completed, the reaction kettle was taken out and cooled to room temperature. Then, the resulting solution was filtered through a 0.22 μm filter membrane to remove unreacted impurities, and the filtered solution was dialyzed using a dialysis bag (3500 Da) for 24 h. Finally, the dialyzed solution was freeze-dried, and the obtained CDs were stored at 4 °C for later use. For experimental use, the CDs were dissolved in deionized water to obtain a CD solution with good dispersibility and uniform particle size.

For complex preparation, a fixed concentration of small interfering RNA targeting Ihh (siIhh) was mixed with varying volumes of the CD solution, resulting in CDs-to-siIhh mass ratios from 1:5 to 3:1. The mixtures were subsequently incubated on ice for 30 min to form CDs/siIhh complexes, which were evaluated using agarose gel electrophoresis. Agarose gel electrophoresis was performed using a 1% agarose gel prepared with a TAE buffer. Nucleic acids were stained with EB (0.5 μg/mL). The quantity of CDs/siIhh loaded per lane was 5 μg, and a 1230 bp RNA ladder. Electrophoresis was conducted in the TAE buffer at a constant voltage of 120 V for 20 min, followed by gel imaging under ultraviolet illumination with Bio-Rad Image Lab software Version 6.1.

### 2.4. In Vitro Transfection Experiment of CDs

ATDC5 cells were seeded in 6-well plates at an appropriate density, approximately 1 × 10^6^ cells per well, and cultured overnight under serum-free conditions. The serum-free medium and original complete medium used in this experiment shared DMEM as the basal medium and were both supplemented with 1% penicillin–streptomycin, and the complete medium was additionally supplemented with 10% fetal bovine serum (FBS). After the cells adhered to the plate and reached the appropriate density, the original medium was carefully aspirated and removed, and the cells were washed 3 times with a sterile PBS solution. Transfection conditions were optimized by adjusting the mass ratios of CDs to GFP plasmid DNA, with the concentration of GFP plasmid DNA maintained at 1.6 μg/mL per well. Subsequently, 2 mL of medium containing complexes of different gene carriers and GFP plasmids was added to each well, including four treatment groups: CDs-1/GFP, CDs-2/GFP, CDs-3/GFP, and CDs-4/GFP. The culture plate was placed in a cell incubator at 37 °C with 5% CO_2_ for further co-culture for 6 h. After 6 h, the original medium was aspirated, and fresh medium was added for continued culture. After 36 h, the medium was discarded. The cells were observed under a fluorescence microscope with the excitation wavelength adjusted to 488 nm, and the green fluorescence intensity was observed and recorded. Three random fields of view were selected per well under the microscope, and ImageJ software Version 1.54g was used to count the number of GFP-positive cells and total cells; transfection efficiency was calculated as the percentage of positive cells, for the evaluation and comparison of transfection efficacy across different treatment groups.

### 2.5. Determination of GFP Transfection Efficiency by Flow Cytometry

The detailed transfection procedure is described in Section 2.4. After transfection, adherent cells were digested with trypsin for 2 min, followed by the addition of serum-containing medium to terminate the reaction. The cells were gently pipetted to obtain a single-cell suspension, which was then transferred to a centrifuge tube, washed, and repeatedly resuspended after centrifugation. Finally, the cell pellet was resuspended in 1 mL of PBS and analyzed using a flow cytometer.

### 2.6. Cytotoxicity Assay

First, 3 × 10^3^ chondrocytes were seeded into a 96-well plate and cultured in fresh medium (DMEM supplemented with 10% FBS). When the cell density reached approximately 80%, the medium was replaced with fresh medium containing PEI, CDs, Lipo2000, or siIhh. Cells were incubated with carriers for 48 h before CCK-8 reading. Then, 10 μL of CCK-8 solution was added to each well, and the cells were incubated for another 30 min at 37 °C with 5% CO_2_. Each group had six replicates (*n* = 6), and the absorbance was measured at 450 nm using a microplate reader. The CCK-8 method was used to detect the survival rate of the above cells. Cell viability was calculated according to the following formula:

Cell viability = [(As − Ab)/(Ac − Ab)] × 100%

In the above formula, As is the absorbance of the experimental well (including cells, medium, CCK-8 solution, and drug solution), Ac is the absorbance of the control well (including cells, medium, CCK-8 solution, and no drug), and Ab is the absorbance of the blank well (including medium, CCK-8 solution, and no cells or drug).

### 2.7. Cellular Uptake

The CDs/siIhh system possesses intrinsic green fluorescence from CDs and red fluorescence from Cy5 labeled on siIhh. To study the intracellular endocytosis process of this nanocomplex, chondrocytes were seeded in a cell culture plate at a density of 1 × 10^5^ cells per well and pre-cultured in DMEM containing 10% fetal bovine serum in an incubator at 37 °C with 5% CO_2_ for 24 h. Subsequently, the cells were treated with fresh medium containing CDs/siIhh, where the concentration of CDs was 5 μg/mL and the corresponding concentration of siIhh was 90 nmol/L, with incubation gradients set at 2 h, 4 h and 6 h. The cellular uptake of CDs/siIhh at each time point was observed using a fluorescence microscope.

### 2.8. Quantitative Real-Time Polymerase Chain Reaction (qRT-PCR) Analysis

The gene expression level was evaluated by quantitative real-time polymerase chain reaction (qRT-PCR). RNA was isolated using TRIzol reagent, and then 1 μg of this RNA was reverse-transcribed into complementary DNA (cDNA) using a PCR kit. The TB Green detection system was used for real-time quantitative PCR amplification. The primer sequences are shown in Table 1. The mRNA abundance of all target genes was normalized using GAPDH as an internal reference, and the relative expression level was calculated using the threshold cycle (Ct) method with the formula R = 2^−ΔΔCt^. To evaluate the results, the obtained data were analyzed using one-way analysis of variance (ANOVA), followed by multiple comparisons, with *p* < 0.05 considered statistically significant.

### 2.9. Western Blot Analysis

Cell lysates were prepared using a RIPA lysis buffer supplemented with protease inhibitors and phosphatase inhibitors. For protein electrophoresis, 10 μL of protein molecular weight marker and 20 μL of each sample were loaded. Proteins in the lysates were separated by 10% SDS-PAGE and subsequently transferred onto PVDF membranes. The membranes were blocked with a TBST buffer containing 5% skim milk for 2 h, followed by incubation with primary antibodies at 4 °C overnight. The primary antibodies used included the following: anti-Ihh antibody (1:1000 dilution), anti-Col2a1 antibody (1:1000 dilution), anti-Smo antibody (1:1000 dilution), anti-Col10a1 antibody (1:1000 dilution), and anti-MMP13 antibody (1:1000 dilution). After incubation, the membranes were incubated with corresponding secondary antibodies at room temperature for 2 h. Protein bands were visualized using the enhanced chemiluminescence (ECL) method and detected with a chemiluminescence imaging system. Densitometric analysis was conducted using ImageJ software, and the relative protein expression levels were normalized with GAPDH as the internal reference protein. Statistical analysis of Western blot data was performed by a one-way analysis of variance (ANOVA) followed by multiple comparisons to assess differences between groups. A *p*-value of less than 0.05 was considered statistically significant.

## 3. Results

### 3.1. Characterization of CDs

Transmission electron microscopy (TEM) images of the four CD samples (Figure S1) revealed that all CDs exhibited uniform spherical morphology. They also showed excellent monodispersity with no observable aggregation. The results demonstrated that CDs-1 had an average particle size of 2.72 nm, CDs-2 of 2.82 nm, CDs-3 of 2.86 nm, and CDs-4 of 2.92 nm. These results collectively confirm that the series of CDs possess a homogeneous nanostructure with highly consistent particle sizes across different samples.

**Figure 1:**
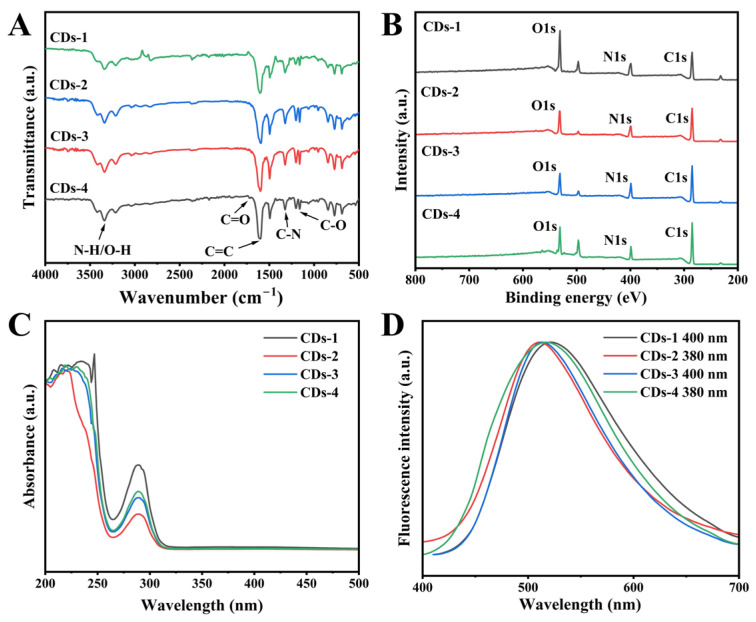
FTIR spectra (**A**), XPS (**B**), UV absorption spectra (**C**), fluorescence spectra (**D**) of CDs.

The surface functional groups of the CDs were studied by FTIR spectra and XPS spectra. As shown in Figure 1A, the peak near 3336 cm^−1^ corresponds to the stretching vibrations of N-H and O-H bonds. The peak at 1700 cm^−1^ indicates the presence of C=O stretching vibrations. Peaks near 1608, 1302, and 1201 cm^−1^ are attributed to the stretching vibrations of C=C, C-N, and C-O bonds, respectively [36]. The XPS results also demonstrated the chemical structure and elemental composition of CDs (Figure 1B). The presence of characteristic C1s (~285 eV), N1s (~399 eV), and O1s (~531 eV) peaks directly confirmed that the main constituent elements of this series of carbon dots were carbon, nitrogen, and oxygen [37]. Peak-fitting deconvolution results (Figure S2) demonstrated the bands of C=C, C-N, C-O, and N-H, which were consistent with the analysis of the FTIR spectra. These results confirm that the CDs’ surface is rich in amino (-NH_2_, -NH-) and carboxyl (-COOH) groups, which are critical functional moieties for subsequent siRNA binding and water dispersion.

The UV absorption spectra of the four CDs are shown in Figure 1C. The absorption in the range of 200–250 nm originates from the π-π* electronic transition of conjugated C=C bonds in the carbon cores, indicating the effective carbonization of their carbon cores. In contrast, the absorption peak in the 250–300 nm range arises from the n-π* transition of surface heteroatom-containing functional groups [38], indicating the presence of abundant surface states on their surface, which are the main factor for the photoluminescence of CDs. The fluorescence spectra of the four CDs were similar (Figure 1D) and mainly covered the green fluorescence. All exhibited excitation-dependent emission behavior (Figure S3), which can be attributed to their analogous surface functional groups. Furthermore, the maximum emission peaks for CDs-1, CDs-2, CDs-3, and CDs-4 were observed at 522 nm, 510 nm, 514 nm, and 517 nm, respectively, under different excitations.

### 3.2. The Impact of Zeta Potential Fluctuations in CDs on Cytotoxicity and Transfection Efficiency

**Figure 2:**
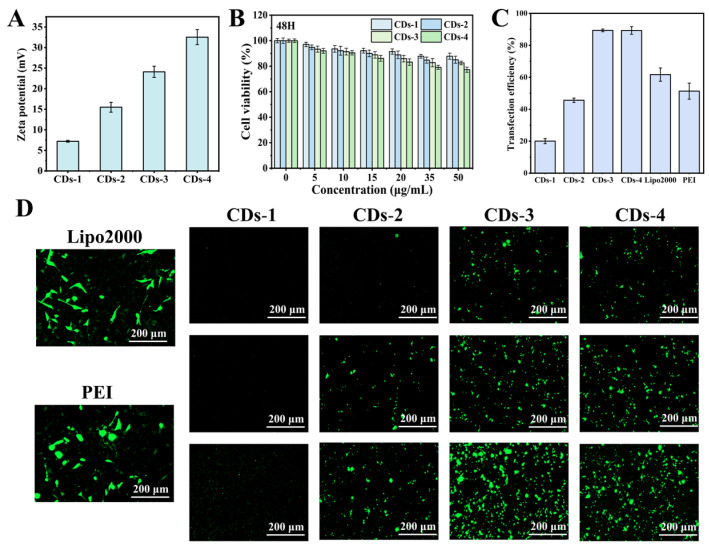
(**A**) The surface zeta potential values of different CDs. (**B**) Cell viability of ATDC5 cells after incubation with different concentrations of CDs for 48 h. (**C**) GFP transfection efficiency by different gene vectors. (**D**) Transfection results of various CDs, Lipofectamine 2000, and PEI. Lipofectamine 2000 and PEI were tested at a mass ratio of 2:1 (vector to GFP); CDs-1/2 were tested at mass ratios of 5:1, 7:1, and 10:1 (top to bottom), and CDs-3/4 at 1:1, 1.5:1, and 2:1 (top to bottom). The scale bar is 200 μm.

The zeta potentials of the four CDs are illustrated in Figure 2A. CDs-1, CDs-2, CDs-3, and CDs-4 all exhibited positive surface charges with zeta potentials of 7.2 mV, 16.7 mV, 25.3 mV, and 34.5 mV, respectively. This increasing trend directly correlates with the rising PEI dosage in the precursor mixture. More amine groups were incorporated into the CDs surface, which enhanced protonation in aqueous solutions and thus elevated zeta potential.

Considering the safety issues associated with potential biomedical applications, toxicity studies were conducted. The viability of ATDC5 cells incubated with different CDs for 48 h is presented in Figure 2B. A dose-dependent pattern was observed for all the CDs, where the cytotoxicity increased with the concentration of the CDs. In addition, for the same concentration, the cytotoxicity gradually increased from CDs-1 to CDs-4, which may be associated with the growing positive potential of the CDs. However, even at a high concentration (50 μg/mL) of CDs, the cell viability remained above 80%, indicating that the CDs possessed good biocompatibility and low cytotoxicity under these experimental conditions.

To evaluate the gene delivery performance of the CDs, green fluorescent protein (GFP) plasmid was selected as the transfection gene model, and the transfection effect of the CDs was compared with commercial Lipo2000 and PEI. Considering the fluorescence of GFP overlaps with CDs in the green channel of the microscope, to reduce the intrinsic fluorescence influence of CDs, a 365 nm ultraviolet lamp was used to irradiate the CDs prior to the experiment by utilizing their photobleaching property (Figure S4). Subsequently, the mass ratios of CDs to GFP plasmid were regulated to screen out the optimal transfection ratio (CDs-1:GFP = 5:1, 7:1, 10:1; CDs-2:GFP = 5:1, 7:1, 10:1; CDs-3:GFP = 1:1, 1.5:1, 2:1; CDs-4:GFP = 1:1, 1.5:1, 2:1). After 36 h post-transfection, the transfection effect was imaged and calculated (Figure 2D). The CDs-3 and CDs-4 groups exhibited the strongest green fluorescent signals with a transfection efficiency approaching 100%, which was significantly higher than that of the CDs-1 and CDs-2 groups. Compared to the transfection efficiency of commercial Lipo2000 and PEI, CDs-3 and CDs-4 groups also presented significant advantage. To determine the GFP transfection efficiency with higher accuracy, we additionally performed flow cytometry assays. The results showed that the transfection efficiencies of CDs-3 and CDs-4 were 90.8% and 87.9%, respectively, as presented in Figure S5, which were consistent with the results in Figure 2D. Because of the low transfection efficiency of CDs-1 and CDs-2, only CDs-3 and CDs-4 were studied in the following work.

### 3.3. Packaging Ability of CDs for siRNA

**Figure 3:**
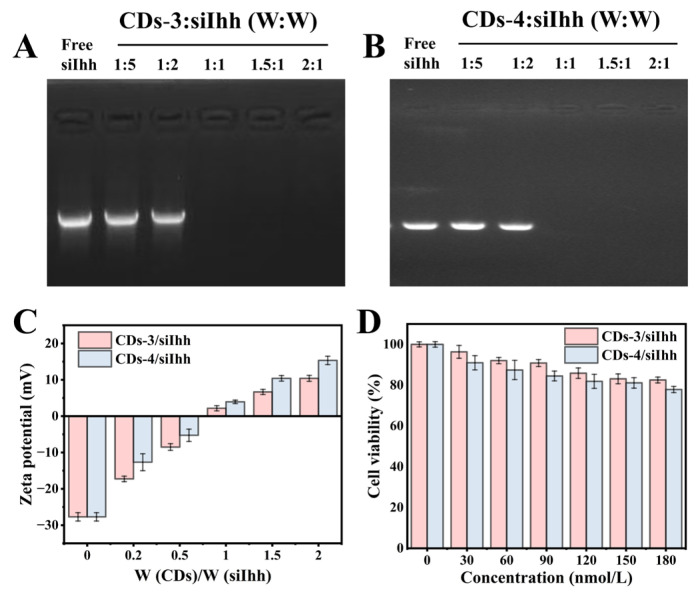
Agar gel results of CDs-3 (**A**) and CDs-4 (**B**) binding to siIhh. (**C**) The zeta potential of CDs-3/siIhh and CDs-4/siIhh complexes at different mass ratios (*n* = 3). (**D**) Cytotoxicity of CDs-3/siIhh and CDs-4/siIhh (the *x*-axis represents the concentration of siIhh).

Based on the high transfection efficiency of the CDs, the nucleic acid encapsulation capability was evaluated using siIhh as a siRNA model. Since the free small interfering RNA siIhh is negatively charged, it migrates toward the positive electrode and thus exhibits a bright band in the gel. From Figure 3A,B, when the mass ratio of CDs to siIhh increased to 1:2 (CDs:siIhh), the migration position of siRNA was almost identical to that of free siRNA. When the mass ratio reached 1:1 (CDs:siIhh), the migration rate of the band slowed down, which was attributed to the increased mass-to-charge ratio of the CDs/siIhh complex. When the mass ratio reached 1.5:1 (CDs:siIhh) and 2:1 (CDs:siIhh), the siIhh band completely disappeared, indicating that siRNA had been fully bound to CDs and no longer migrated toward the positive electrode. On the other hand, as the mass ratio of CDs to siRNA increased, the zeta potential of the CDs/siIhh complex system accordingly changed from negative to positive, which also confirmed the binding of free siRNA to CDs. At a mass ratio of 1.5:1 (CDs:siIhh), although the band disappeared entirely, the zeta potential results suggested that the stability of the complex might be slightly lower. Thus, based on the above results, a mass ratio of CDs to siRNA of 2:1 was selected for the formation of stable complexes, which is crucial for the successful delivery of siRNA.

The cytotoxicity of CDs/siIhh complexes was further assessed (Figure 3C,D). Both the cell viability treated with CDs-3/siIhh and CDs-4/siIhh were above 80%, even at a high concentration (180 nmol/L). In comparison, the viability of the CDs-3/siIhh group was higher than that in the CDs-4/siIhh group at the same concentration. Considering the transfection efficiency and cytotoxicity of bare CDs and CDs/siIhh complexes together, CDs-3 was regarded as the optimal gene vector, and the CDs-3/siIhh system was adopted for the subsequent therapeutic processes. Ultimately, the concentration of 90 nmol/L for the CDs-3/siIhh system, at which the cell viability was approximately 90%, was selected in the subsequent study.

### 3.4. Cellular Uptake of CDs-3/siIhh Complex

The CDs-3/siIhh complex was further characterized, with its particle size determined to be 12 nm (Figure S6). As a core parameter of gene delivery systems, an optimal particle size facilitates the vector to cross the cell membrane and exert gene regulatory effects. This result also confirms that CDs-3 can form stable, non-aggregated nanocomplexes with siIhh, laying a critical foundation for the performance stability of the delivery system in subsequent experiments.

**Figure 4:**
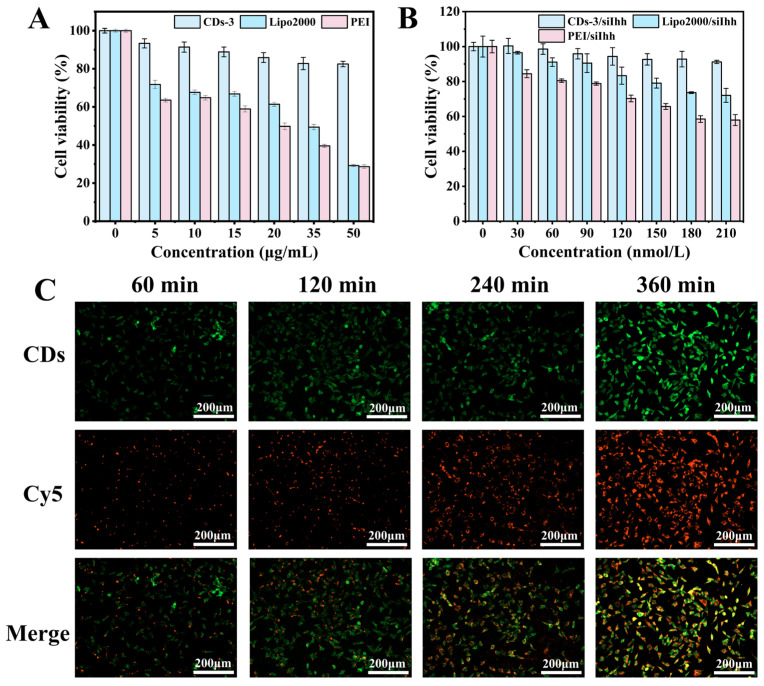
(**A**) Cytotoxicity of CDs-3, Lipo2000, and PEI. (**B**) Cytotoxicity of CDs-3/siIhh, Lipo2000/siIhh, and PEI/siIhh (the *x*-axis represents the concentration of siIhh). (**C**) Cellular uptake of CDs/siIhh with different incubation times. The scale bar is 200 μm.

The optimal gene vector CDs-3 was further evaluated by comparative experiments with commercial carriers Lipo2000 and PEI (Figure 4A,B). The cell viability of CDs-3 was obviously superior to Lipo2000 and PEI. When using a concentration above 20 μg/mL, the cell viabilities of Lipo2000 and PEI groups were almost down to 50%, exhibiting significant cytotoxicity. For the systems carrying gene siIhh, the cell viability of the CDs-3/siIhh group was also significantly greater than the Lipo2000/siIhh or PEI/siIhh groups. These results indicate that both the bare CDs and the CDs-3/siIhh complex possess better biocompatibility.

To investigate the in vitro cellular uptake of the CDs-3/siIhh complex, the Cy5 red fluorescent dye was conjugated to siIhh to track the cellular delivery process of siIhh at different incubation times (Figure 4C). After 60 min of incubation, distinct green fluorescence was observed in the cytoplasmic and nuclear regions of cells, while only extremely weak red signals were detectable. This indicated that the CDs-3/siIhh complex had successfully penetrated the cell membrane and entered the cell interior. As the incubation time extended to 240 min, the brightness of both green and red fluorescence increased with further expanded coverage, and the cellular delivery amount of the CDs-3/siIhh complex gradually increased. When the incubation time reached 360 min, the fluorescence brightness in both channels reached a saturated state. The fluorescence in the merged channel was highly overlapped and covered almost all observed cells, demonstrating that the CDs-3/siIhh complex had been fully internalized by the cells and achieved sufficient intracellular distribution. These experimental results demonstrated that the in vitro cellular uptake of the CDs-3/siIhh complex exhibited a time-dependent process. Quantitative analysis of the Cy5 red fluorescence signal revealed a continuous increase in fluorescence intensity with prolonged incubation time (Figure S7), further validating the time-dependent nature of the in vitro cellular uptake of the CDs-3/siIhh complex. CDs-3 itself possessed excellent cell membrane penetration ability, enabling it to enter cells rapidly and diffuse extensively within the cytoplasm. Meanwhile, the stable binding of CDs-3/siIhh ensured the sustained delivery of the complex, and the low cytotoxicity of CDs-3/siIhh maintained the normal physiological state of cells, thereby providing favorable conditions for the gradual diffusion and ultimate sufficient distribution of the complex in the cells.

### 3.5. Regulatory Effects of CDs/siIhh at Gene Level

To explore the regulatory effects of the CDs-3/siIhh complex, quantitative real-time polymerase chain reaction (qRT-PCR) was employed to detect the expression levels of cartilage-specific markers [type II collagen alpha 1 chain gene (Col2a1), type X collagen alpha 1 chain gene (Col10a1)], matrix metalloproteinase 13 (MMP13), Indian Hedgehog protein (Ihh), and its downstream protein (Smo).

**Figure 5:**
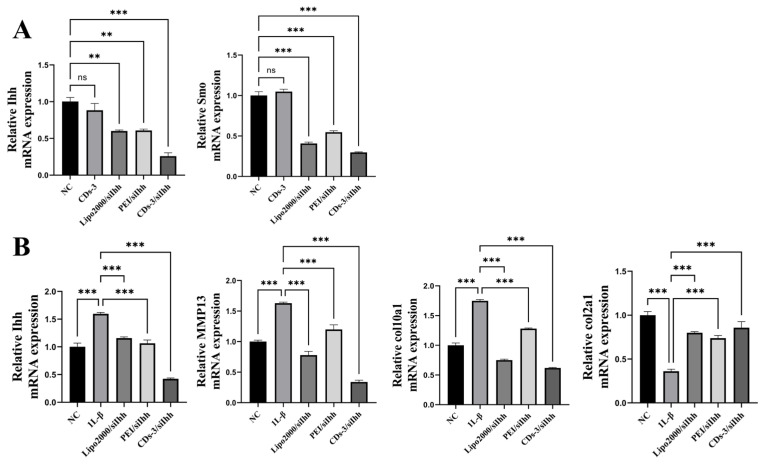
(**A**) Relative mRNA levels of genes (Ihh, Smo). (**B**) Relative mRNA levels of cartilage-specific markers (Col2a1 and Col10a1) and OA-related genes (MMP13, Ihh). The data are presented as mean ± SD (*n* = 3). ns *p* > 0.05, ** *p* < 0.01, *** *p* < 0.001.

As depicted in Figure 5A, in normal ATDC5 cells, the CD group showed no significant difference from the NC (negative control) group, indicating that CDs themselves do not nonspecifically interfere with the Ihh signaling pathway in normal cells. In contrast, the mRNA expressions of Ihh and Smo in the Lipo2000/siIhh, PEI/siIhh, and CDs-3/siIhh groups were all significantly downregulated, with the CDs-3/siIhh group exhibiting the most remarkable effect. This indicates that the delivery efficiency of CDs for siIhh is superior to that of commercial vectors Lipo2000 and PEI, probably due to their moderate zeta potential that balances the binding affinity for siRNA and cellular uptake efficiency, thus avoiding the adverse effects of excessive carrier aggregation or cytotoxicity on transfection.

Cells were treated with 20 ng/mL IL-1β for 48 h to induce an inflammatory cell model of osteoarthritis (OA). After IL-1β stimulation, the expressions of Ihh, MMP13, and Col10a1 in the NC group were significantly increased, while the expression of normal cartilage matrix marker Col2a1 was significantly decreased, successfully establishing an inflammatory cell model of OA (Figure 5B). On this basis, the Lipo2000/siIhh, PEI/siIhh, and CDs-3/siIhh groups all exhibited reverse regulation of the aforementioned genes Ihh, MMP13, and Col10a1 were significantly downregulated, and Col2a1 was significantly upregulated. Furthermore, the CDs-3/siIhh group showing the most significant regulatory effect among all groups. This result highlights the advantages of CDs in the pathological inflammatory microenvironment. Their low toxicity and strong cell permeability enable efficient siIhh delivery, thereby maximally blocking the pro-inflammatory and pro-degenerative effects of the Ihh pathway and providing a carrier solution with both efficiency and safety for OA gene therapy.

### 3.6. Deciphering the Regulatory Effects of CDs-3/siIhh at the Protein Level

**Figure 6:**
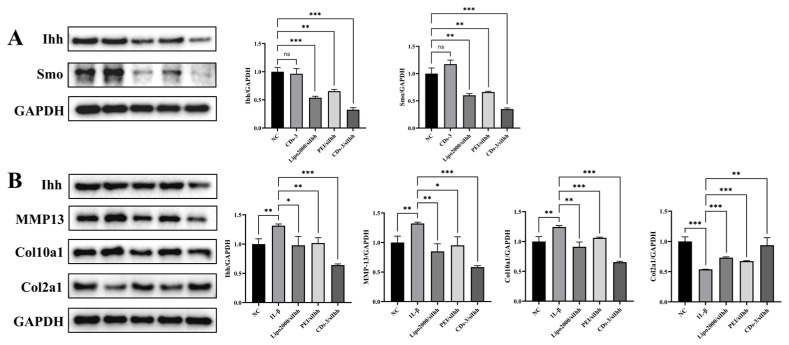
(**A**) Expression levels of Ihh and its downstream protein Smo. (**B**) Protein expression levels of Ihh, MMP13, Col10a1, and Col2a1. The data are presented as mean ± SD (*n* = 3). ns *p* > 0.05, * *p* < 0.05, ** *p* < 0.01, *** *p* < 0.001.

As shown in Figure 6A, a Western blot analysis was carried out to give an in-depth understanding of the regulatory effects of the CDs-3/siIhh system at the protein functional level. There was no significant difference between the NC group and the CD group, indicating that CDs-3 also have excellent biocompatibility at the protein level and will not nonspecifically interfere with the protein expression regulation network of normal cells, providing direct evidence at the protein level for their “safety” as gene vectors. Compared with the Lipo2000/siIhh and PEI/siIhh groups, the Ihh and Smo proteins in the CDs-3/siIhh group were significantly more downregulated, reflecting the efficient delivery of siIhh by CDs-3 and, more importantly, that the siIhh mediated by CDs can stably inhibit the translation process of target proteins.

After IL-1β treatment, the protein expressions of MMP13, Ihh, and Col10a1 in the NC group were significantly increased, while the expression of Col2a1 was significantly decreased (Figure 6B). After treatment with Lipo2000/siIhh, PEI/siIhh, and CDs-3/siIhh, the aforementioned proteins showed reverse regulation: MMP13, Ihh, and Col10a1 were significantly downregulated, and Col2a1 was significantly upregulated, with the CDs-3/siIhh group showing the most significant regulatory effect. This indicates that the low toxicity and moderate size of CDs enable them to maintain efficient transfection in inflamed stressed cells, ensuring the precise delivery of siIhh to target cells and blocking the pro-inflammatory and pro-degenerative cascade of the Ihh pathway at the protein level.

The high concordance between WB and qRT-PCR results confirmed that CDs-3 could effectively mediate the holistic regulation of target genes throughout the entire process from transcription to translation. Within the observation time window of the in vitro experimental system established in this study, the inhibition of target gene expression exhibited distinct synchrony at the transcriptional and translational levels, and no apparent signs of discordance between mRNA and protein expression were detected. This finding lays a solid in vitro molecular foundation for subsequent in-depth investigations into osteoarthritis-related gene regulation.

## 4. Discussion

This study successfully developed a positive-potential CD-based siRNA delivery system (CDs/siIhh) for gene therapy. Precursor ratios were adjusted to synthesize CDs with varying positive potentials and screen the formulation with the most suitable surface potential for nucleic acid delivery. Characterization confirmed that the CDs possessed good dispersibility, surface amino/carboxyl groups, and positive potentials ranging from 7.23 mV to 34.5 mV. These properties laid the structural foundation for efficient siRNA binding and stable aqueous dispersion of the CDs. Among them, CDs-3 (25.3 mV) showed optimal performance. It had low cytotoxicity, with cell viability >80% even at high concentrations. It also exhibited high transfection efficiency, nearly 100% when delivering GFP plasmid. Moreover, it could bind siIhh efficiently at a 2:1 mass ratio. In vitro experiments demonstrated that the CDs-3/siIhh delivery system efficiently downregulated Ihh and downstream Smo expression, upregulated Col2a1, and reduced Col10a1 and MMP13 levels. CDs with an optimal positive potential (25.3 mV) possessed excellent biocompatibility and gene delivery capacity. They were markedly superior to commercial vectors Lipo2000 and PEI. These commercial vectors often face a trade-off between transfection efficiency and cytotoxicity in practical applications. This work developed a novel CD-based gene delivery vector with favorable safety and delivery efficiency, which holds potential application value for relevant gene regulation research.

All functional validation experiments in this paper were conducted in a pure in vitro cell culture system under strictly controlled conditions. While this ensured the reliability of the obtained results, the conclusions are only limited to the in vitro cellular level, without further verification in complex physiological fluid environments such as synovial fluid and serum, as well as in tissue and animal models that better mimic the in vivo setting. In subsequent studies, we will further expand the experimental system to investigate the performance of this delivery system under more physiologically relevant conditions, thereby completing a comprehensive validation of the gene regulatory effects mediated by CDs-3.

## Figures and Tables

**Table 1:** The primer sequences used for qRT-PCR.

Gene	Forward Primer	Reverse Primer
GAPDH	CATCACTGCCACCCAGAAGACTG	ATGCCAGTGAGCTTCCCGTTCAG
Ihh	CGGCTTCGACTGGGTGTATTAC	AGGAAAGCAGCCACCTGTCTTG
Col2a1	GCTGGTGAAGAAGGCAAACGAG	CCATCTTGACCTGGGAATCCAC
Col10a1	GTACCAAACGCCCACAGGCATA	GGACCAGGAATGCCTTGTTCTC
MMP13	GATGACCTGTCTGAGGAAGACC	GCATTTCTCGGAGCCTGTCAAC
Smo	GAGGCTACTTCCTCATCAGAGG	GCTGAAGGTGATGAGCACAAAGC

## Data Availability

The data that support the findings of this study are available from the corresponding authors upon reasonable request.

## Supplementary Materials

The following supporting information can be downloaded at: https://www.mdpi.com/article/10.3390/nano16030169/s1, Figure S1: Transmission electron microscopy (TEM) images and particle size distribution statistics of different carbon dots (CDs). (A-D) TEM images of CDs-1 to CDs-4 (scale bar: 20 nm), respectively. Figure S2: High-resolution XPS spectra of CDs (A) C1s, (B) O1s, and (C) N1s peaks of CDs. Figure S3: Fluorescence spectra of CDs. Figure S4: Photobleaching results of CDs. Figure S5: Flow cytometry scatter plots. A: Blank control (Con, 0.15%); B-E: CDs-1 to CDs-4 vector groups (25.3%, 48.9%, 90.8%, 87.9%); F-G: Traditional vector controls (Lipo2000: 54.8%; PEI: 64.7%). Figure S6: Transmission electron microscopy (TEM) image and particle size distribution statistics of CDs-3/siIhh complex (mass ratio 2:1). Figure S7: Quantitative analysis of cellular uptake of CDs-3/siIhh complexes.
