# Supplementary material for: Fine-Tuning Positive-Surface-Charge Carbon Dots for High-Efficiency and Low-Cytotoxicity Gene Delivery
Source: Nanomaterials (Basel). 2026 Jan 26;16(3):169. doi: 10.3390/nano16030169 (PMC12899058; doi:10.3390/nano16030169)
Supplement: Supplementary file 1 [file nanomaterials-16-00169-s001.zip › nanomaterials-4045732-supplementary.pdf]

Supporting Information

Fine-Tuning Positive-Surface-Charge Carbon Dots for High-Efficiency and Low-Cytotoxicity Gene Delivery

Shuo Zhang, Yangming Zhou, Qi Zhang, Juanjuan Xue, Ruijie Li, Tao Liu, Qianqian Duan * and Shengbo Sang *

Shanxi Key Laboratory of Artificial Intelligence & Micro Nano Sensors, School of Integrated Circuit, Taiyuan University of Technology, Taiyuan 030024, China; zshuo0123@163.com (S.Z.); 2023521518@tyut.edu.cn (Y.Z.); 13593432945@163.com (Q.Z.); xuejuanjuan@tyut.edu.cn (J.X.); 2023521510@tyut.edu.cn (R.L.); wang1577616430@163.com (T.L.)

* Correspondence: duanqianqian@tyut.edu.cn (Q.D.); sunboa-sang@tyut.edu.cn (S.S.)

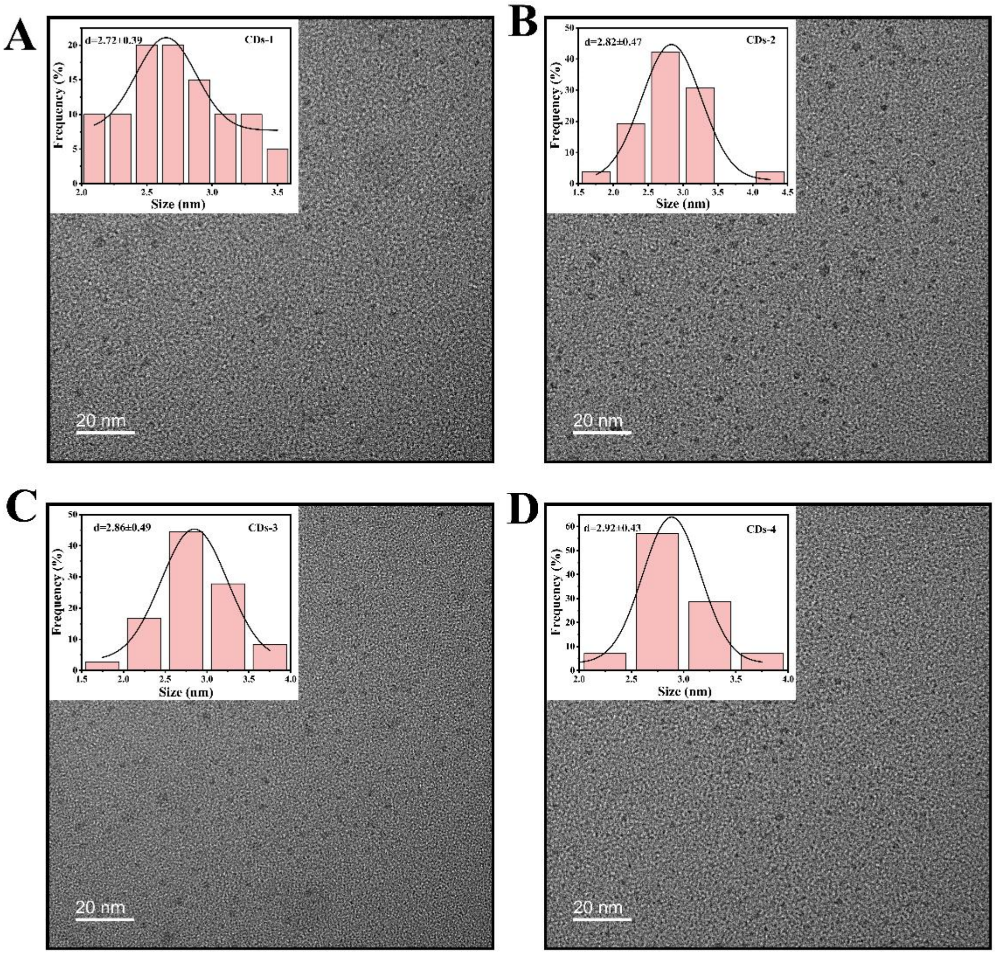

Figure S1. Transmission electron microscopy (TEM) images and particle-size distribution statistics of different carbon dots (CDs). (A-D) TEM images of CDs-1 to CDs-4 (scale bar: 20 nm), respectively.

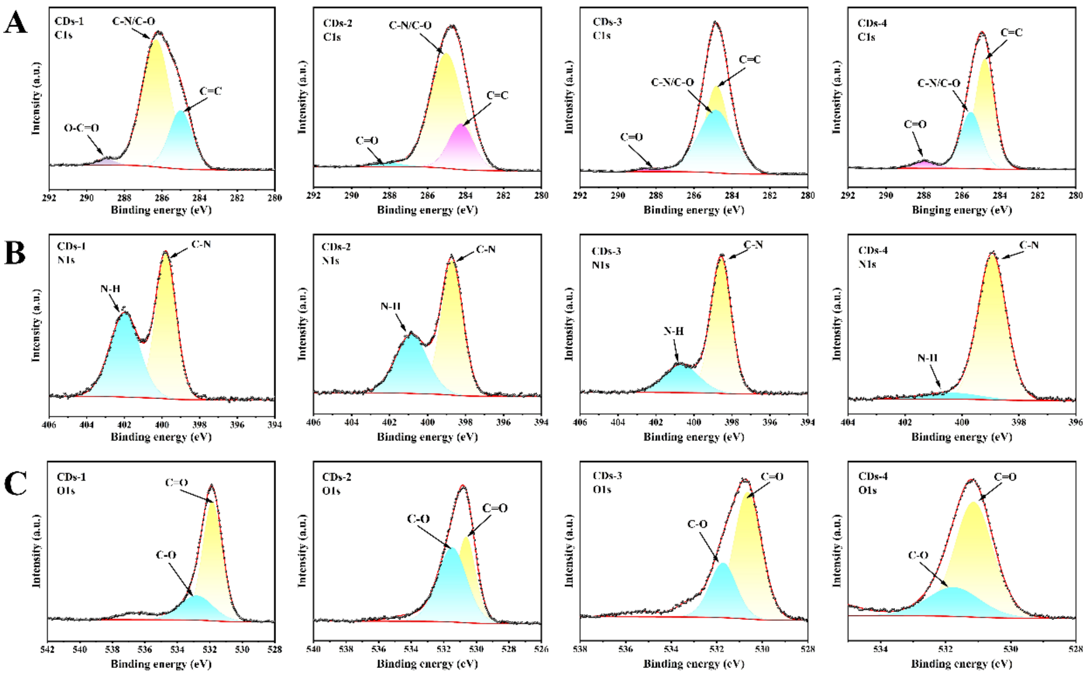

Figure S2. High-resolution XPS spectra of CDs (A) C1s, (B) O1s, and (C) N1s peaks of CDs.

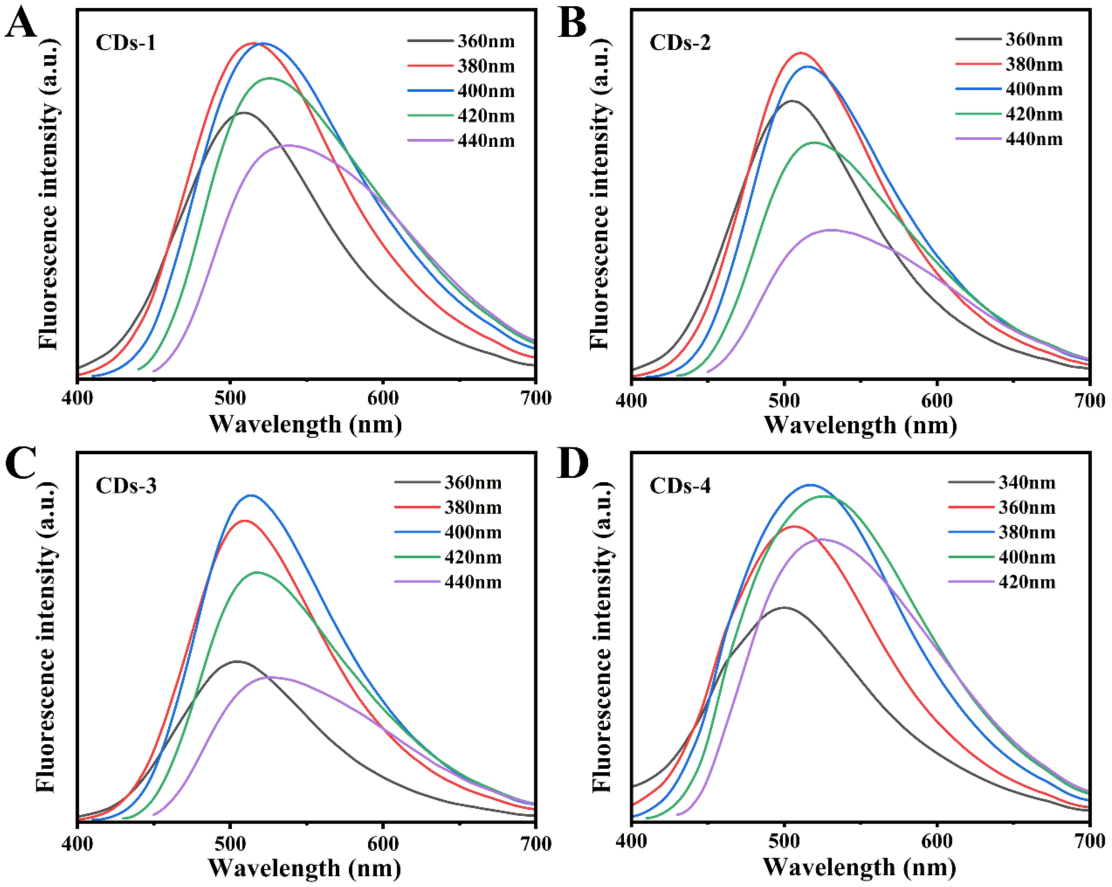

Figure S3. Fluorescence spectra of CDs

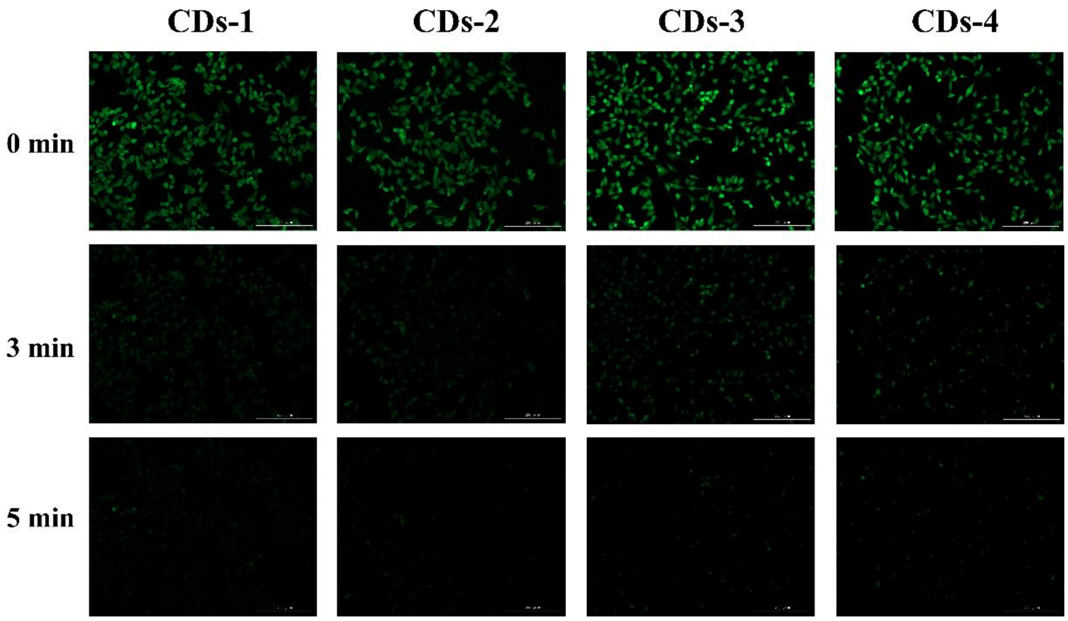

Figure S4. Photobleaching results of CDs

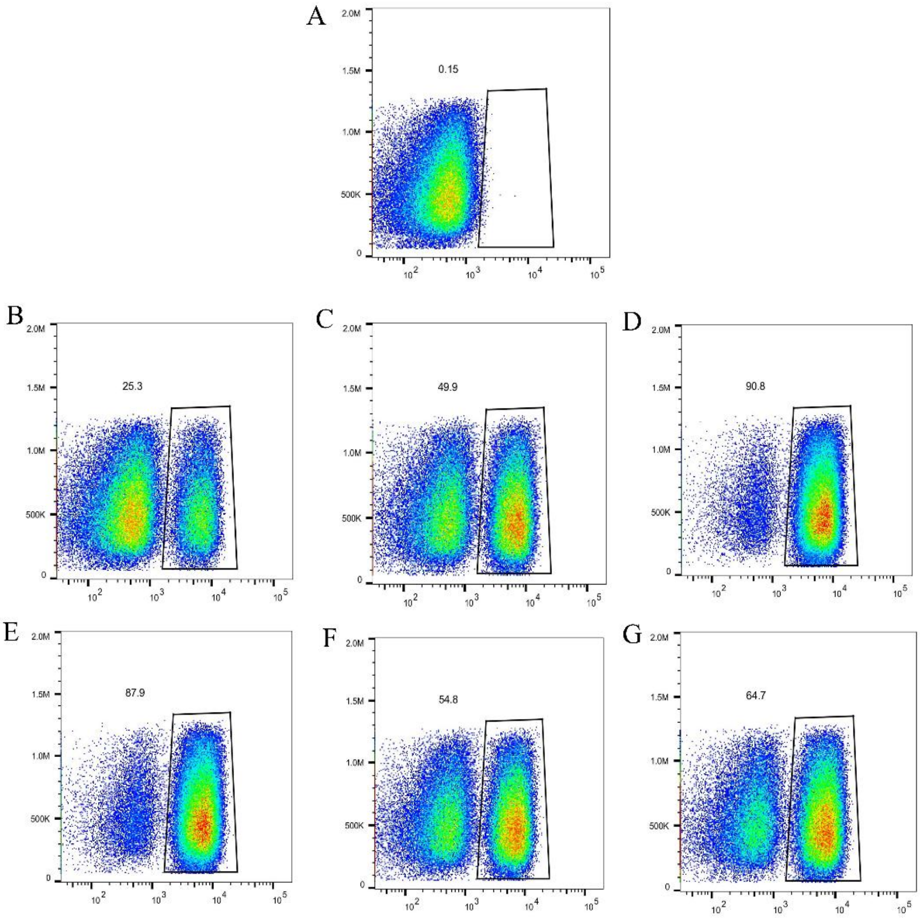

Figure S5. Flow cytometry scatter plots. A: Blank control (Con, 0.15%); B-E: CDs-1 to CDs-4 vector groups (25.3%, 48.9%, 90.8%, 87.9%); F-G: traditional vector controls (Lipo2000: 54.8%; PEI: 64.7%).

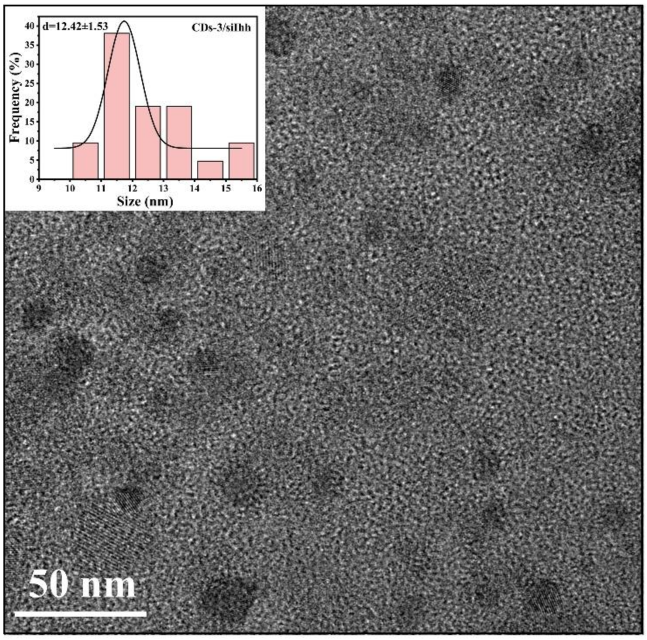

Figure S6. Transmission electron microscopy (TEM) image and particle-size distribution statistics of CDs-3/siIhh complex (mass ratio 2:1)

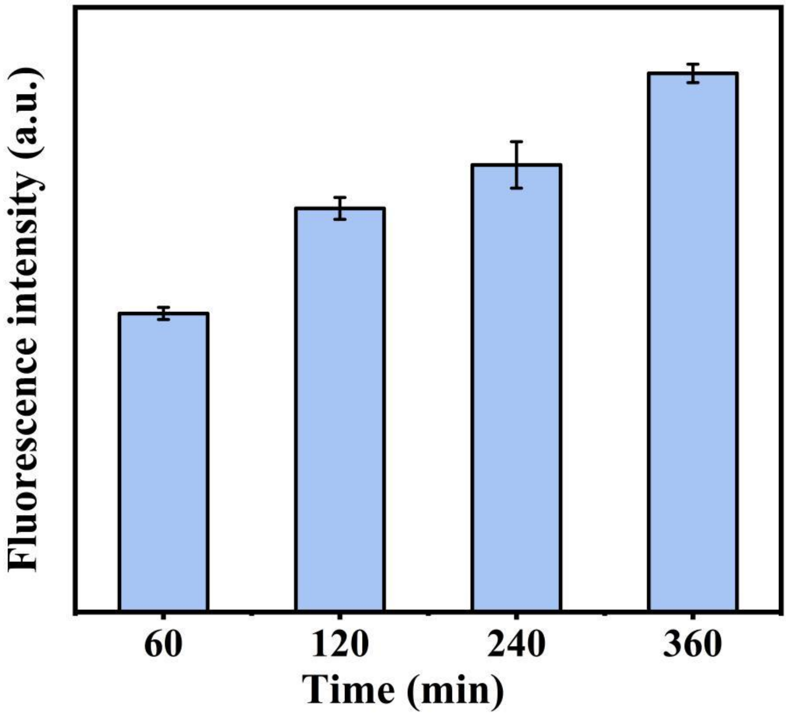

Figure S7. Quantitative analysis of cellular uptake of CDs-3/siIhh complexes
